# Perioperative Immunonutrition and Postoperative Recovery Patterns in Oral Squamous Cell Carcinoma Surgery: A Prospective Observational Cohort Study

**DOI:** 10.1155/jnme/4971173

**Published:** 2026-10-06

**Authors:** F. Ferragina, Y. Ferro, I. Barca, A. R. Sottile, R. Pujia, A. Arrotta, T. Montalcini, M. G. Cristofaro

**Affiliations:** ^1^ Department of Experimental and Clinical Medicine Magna Graecia University Catanzaro Italy unicz.it; ^2^ Department of Medical and Surgical Sciences Magna Graecia University of Catanzaro Catanzaro Italy unicz.it; ^3^ Department of Neurosciences, Reproductive and Odontostomatological Sciences Federico II University Naples Italy unina.it; ^4^ Department of Health Science Magna Graecia University Catanzaro Italy unicz.it

**Keywords:** hospital stay reduction, muscle strength, oral squamous cell carcinoma, perioperative immunonutrition

## Abstract

**Background:**

Malnutrition and sarcopenia negatively affect outcomes in patients undergoing surgery for oral squamous cell carcinoma (OSCC). Immunonutrition has been proposed to improve perioperative recovery, but evidence remains limited, particularly in oral cancer surgery. The objective of this study was to evaluate postoperative weight changes, muscle status, complications, and hospital stay in patients with OSCC who received a perioperative immune‐nutritional protocol.

**Methods:**

We conducted a single‐center prospective observational cohort study including 22 patients surgically treated for OSCC between January 2022 and January 2024. All patients received perioperative immunonutrition administered orally or via nasogastric tube at four timepoints (T0: preoperative; T1: immediate postoperative; T2: discharge; T3: 30 days). All patients underwent anthropometric measurements, muscle strength assessment, and bioelectrical impedance analysis (muscle mass, phase angle, and total body water). All evaluations were performed at each study timepoint.

**Results:**

The study population consisted of 62% females, with a mean age of 65 ± 10 years, and 17% of patients presented clinically relevant nutritional risk at baseline. Patients experienced a mean weight loss of 2.1 kg during the early postoperative hospitalization period, followed by a partial recovery of 1.2 kg within 30 days (*p* < 0.001). Phase angle and handgrip strength remained stable throughout the follow‐up, indicating preserved cellular integrity and muscle function. Wound dehiscence occurred in 38% but healed without major sequelae. The average length of hospital stay was 13.8 ± 6.9 days. Although the results are lower than those reported in the literature on similar OSCC cases, caution is warranted when comparing due to differences in design and perioperative management.

**Conclusions:**

In patients undergoing surgery for OSCC, the perioperative immune‐nutritional protocol was associated with minimal postoperative weight loss and stable functional muscle status. Additionally, the postoperative recovery patterns in this cohort were favorable, and the complication rates were low.

**Clinical Implications:**

Integrating immunonutrition into enhanced recovery (ERAS) pathways may optimize surgical outcomes, reduce hospitalization costs, and improve patient quality of life.

## 1. Introduction

Oral squamous cell carcinoma (OSCC) is among the most common malignancies of the head and neck, with a significant global impact in terms of incidence, morbidity, and mortality. Despite advancements in surgical techniques, radiotherapy, and systemic therapy, long‐term survival remains unsatisfactory, and postoperative complications continue to impact both prognosis and quality of life [1–4].

Nutritional status has been demonstrated to exert a substantial influence on the clinical progression of patients diagnosed with OSCC. Malnutrition has been documented in 30%–50% of patients diagnosed with head and neck cancers, and it is associated with reduced muscle mass and impaired muscle function, delayed wound healing, an increased risk of postoperative infections, prolonged hospital stay, and reduced tolerance to oncologic treatments [5–9]. A multitude of mechanisms contribute to nutritional decline, including tumor‐related dysphagia, pain, reduced appetite, and treatment‐related toxicities [10, 11]. Reduced muscle mass and impaired muscle function have emerged as negative prognostic markers, correlating with higher complication rates and reduced survival [12–17]. Several studies have clarified the biological and clinical mechanisms underlying this association. The alterations in staging and grading systems highlight the growing relevance of host‐related factors, including body composition, in the prognosis of OSCC [12]. A substantial body of research has demonstrated that reduced muscle mass and sarcopenia are associated with poorer surgical outcomes, increased postoperative complications, and reduced overall and disease‐free survival [13]. The etiology of these effects is likely mediated by cancer‐related inflammation, oxidative stress, metabolic dysregulation, and malnutrition, all of which have been shown to contribute to muscle wasting and impaired treatment tolerance [14–16]. In addition, the occurrence of dysphagia has also been found to worsen the nutritional deficiencies and muscle impairment commonly seen in head and neck cancer patients, leading to poor quality of life and shortened lifespan [17].

To counteract these effects, nutritional support during the perioperative period has become an integral part of multimodal oncologic care. Immunonutrition, a specialized formula enriched with arginine, omega‐3 fatty acids, nucleotides, and antioxidants, has been proposed to modulate immune response, attenuate systemic inflammation, and enhance wound healing. Several randomized controlled trials and meta‐analyses have demonstrated that perioperative immunonutrition can reduce infectious complications, shorten hospital stay, and improve overall outcomes for patients undergoing major gastrointestinal and head and neck cancer surgery. These benefits appear particularly relevant in the context of OSCC, where baseline nutritional impairment and treatment‐related inflammation are highly prevalent [4, 7]. Systematic reviews and randomized controlled trials have demonstrated that perioperative immunonutrition, enriched with specific substrates such as arginine, omega‐3 fatty acids, and nucleotides, modulates immune response, attenuates inflammatory pathways, and improves wound healing, thereby reducing postoperative infectious complications and pain [5, 8, 18]. In patients receiving multimodal treatments, including surgery and chemoradiotherapy, immunonutrition has also been associated with improved treatment tolerance and a lower incidence and severity of therapy‐related toxicities, such as oral mucositis [6, 9]. Furthermore, nutritional prehabilitation strategies incorporating immunonutrition have been shown to optimize perioperative status, reduce hospital stays, and improve overall clinical outcomes [11, 19].

However, most of the available evidence derives from gastrointestinal or mixed head and neck cancer cohorts, while data specifically addressing oral cavity cancer remain scarce. Although recent guidelines recommend the use of immunonutrition as part of enhanced recovery (ERAS) programs, its impact in OSCC surgery has not been systematically evaluated. This represents a critical knowledge gap in perioperative nutritional management for oral cancer patients.

The present prospective observational study was designed to evaluate the effects of perioperative immunonutrition on postoperative weight loss, muscle preservation, complication rate, and hospital length of stay (LOS) in patients undergoing surgery for OSCC.

## 2. Materials and Methods

### 2.1. Study Design and Setting

This prospective observational cohort study was conducted at the Maxillofacial Surgery Unit of the University Hospital of Catanzaro, Italy, in collaboration with the Clinical Nutrition Unit. The study period extended from January 2022 to January 2024. All patients were managed according to a standardized perioperative care protocol that included nutritional assessment and individualized immunonutritional support.

All procedures were conducted in accordance with the principles of the Declaration of Helsinki. The study was conducted in accordance with the Regional Ethics Committee’s guidelines (protocol n. 278/2024), and all participants provided written informed consent before enrollment.

### 2.2. Participants

Patients were eligible for the study if they met the following inclusion criteria: a histologically confirmed diagnosis of OSCC; patients at least 18 years of age who had signed an informed consent form; patients with complete clinical documentation; patients with the ability to complete the nutritional protocol and attend follow‐up visits; patients who underwent a preoperative diagnostic workup and staging according to the 8th edition of the AJCC TNM classification [20, 21]. The clinical and pathological staging of the subjects was determined based on primary tumor size combined with depth of invasion (DOI, in millimeters) for category T and the number, size, and presence of extranodal extension (ENE) of metastatic lymph nodes for category N, in accordance with updated criteria for oral cavity subsites.

The exclusion criteria were as follows: medical history of another solid or hematologic malignancy within the past 5 years and a diagnosis other than squamous cell carcinoma; evidence of metastatic disease; prior neoadjuvant radio‐ or chemotherapy; patients with severe nutritional impairment who require individualized intensive nutritional support were excluded from the study to maintain homogeneity of perioperative management; and patients unable to undergo bioimpedance analysis due to technical or patient‐related factors.

### 2.3. Preoperative Assessment and Surgical Treatment

A meticulous clinical evaluation was conducted for all patients, encompassing a thorough medical history with a particular focus on comorbidities (e.g., diabetes, cardiovascular disease, and hypertension), medication use, and tumor‐related symptoms such as pain, dysphagia, bleeding, or changes in speech. The physical examination entailed a thorough evaluation of the primary lesion, encompassing its dimensions, mobility, and tenderness. Additionally, the examination focused on the cervical lymph nodes and the functionality of adjacent structures. The diagnostic workup entailed histological confirmation by incisional biopsy, as well as blood tests. Additionally, first‐ and second‐level imaging procedures were conducted, including ultrasound, CT, and MRI scans. These factors were essential for accurate estimation of the clinical T category (particularly tumor thickness and DOI) and regional nodal disease (N category) before surgery. Definitive staging was subsequently confirmed or updated by postoperative histopathological analysis of the resected specimen and neck dissection samples, verifying microscopic margins, exact DOI, and ENE status.

Patients who met the inclusion criteria were subjected to surgical procedures with the objective of a curative outcome. These surgical procedures entailed the wide local excision of the primary tumor, accompanied by the establishment of adequate oncologic margins. Depending on the stage and location of the tumor, a selective neck dissection was performed. Reconstructive procedures encompassed local or regional flap reconstruction to restore both function and aesthetics. A nasogastric tube (NGT) was placed in patients with a high risk of postoperative feeding difficulties. The purpose of this procedure was twofold: first, to protect the intraoral wounds of the patients, and second, to ensure adequate nutrition.

### 2.4. Nutritional and Functional Assessment

A four‐point evaluation was conducted for each patient. The following time points were identified:

- T0: 15 days before surgery (baseline).
- T1: immediate postoperative (1–2 days).
- T2: hospital discharge.
- T3: 30 days postoperatively.

At each designated time point (T0, T1, T2, and T3), patients underwent a standardized nutritional evaluation that included the following components:

- Anthropometry: body weight, body mass index (BMI, kg/m^2^), and percentage of weight change from baseline.
- Nutritional status was assessed using the Patient‐Generated Subjective Global Assessment (PG‐SGA). According to the original validation study, the PG‐SGA score was used as a nutritional risk screening tool rather than a direct diagnostic classification of malnutrition severity. Higher scores indicate a greater need for nutritional intervention and symptom management. In the present study, a PG‐SGA score ≥ 4 was considered indicative of clinically relevant nutritional risk requiring nutritional intervention, while no patients presented scores suggestive of critical nutritional risk [22].
- Muscle function: evaluated by handgrip strength (Jamar dynamometer, mean of three attempts). For each strength test, participants performed three maximal isometric contractions, each lasting 3 s. The mean value of the three attempts was taken as the final score.
- Muscle strength was evaluated according to EWGSOP2 (European Working Group on Sarcopenia in Older People) recommendations using handgrip dynamometry. The reduction in muscle strength was taken as a sign of probable sarcopenia; however, since muscle quantity and quality were not assessed using validated diagnostic tools for sarcopenia confirmation, the term “probable sarcopenia” was adopted throughout the manuscript [23, 24].
- Body composition: It was analyzed via bioelectrical impedance analysis (BIA) (Akern BIA 101, Florence, Italy), including skeletal muscle mass, phase angle, and total body water. A phase angle < 5.0° was considered indicative of impaired cellular integrity [25].
- Energy requirements were estimated via predictive equations (Harris–Benedict) adjusted for stress factors.

### 2.5. Immuno‐Nutritional Protocol

All patients received perioperative immunonutrition with a high‐protein (6.7–7.6 g/portion), omega‐3, and polyunsaturated fatty acid–enriched (1.1–1.3 g/portion) formula, providing approximately 1.3–1.4 kcal/mL, with a daily intake of 200–250 mL per portion.

The standardized regimen was as follows:

- Preoperative phase (T0): 2 bricks/day orally for 7–14 days before surgery;
- Immediate postoperative phase (T1): 2‐3 bricks/day via NGT until oral intake was tolerated;
- Discharge phase (T2–T3): oral continuation up to 30 days postsurgery, adjusted according to nutritional needs and tolerance.

For patients receiving enteral nutrition via a NGT, the amount of nutritional support was gradually increased according to the patient’s clinical tolerance and gastrointestinal function until the estimated caloric requirements were met. At that point, a transition to oral feeding was made, when medically appropriate.

Patient adherence to the immunonutritional protocol was clinically monitored at each follow‐up visit by the clinical nutrition team through patient/caregiver reporting and estimation of consumed formula portions. However, no formal quantitative adherence scoring system or patient‐level compliance analysis was performed.

### 2.6. Outcomes and Statistical Analysis

The primary outcomes of the study were postoperative changes in body weight and muscle mass between timepoints. Secondary outcomes included the occurrence of postoperative complications, such as surgical site infection, wound dehiscence, fistula formation, and cranial nerve dysfunction, as well as hospital LOS. Descriptive statistics were expressed as mean ± standard deviation (SD) for continuous variables and as absolute values and percentages for categorical variables. For graphical representation, continuous variables are presented as mean values with error bars indicating the standard error of the mean (SEM). Paired Student’s *t*‐tests were used to evaluate differences between time points (T0–T1, T1–T2, and T2–T3). Statistical significance was set at *p* < 0.05. Analyses were performed using IBM SPSS Statistics Version 31.0 (IBM Corp, Chicago, IL, USA).

## 3. Results

Initially, a total of 24 subjects were included in the screening phase of the study; 22 (91.7%) of these individuals subsequently met the established inclusion criteria and were included in the final data set, having also completed the postintervention follow‐up phase. The cohort comprised 12 females (54.54%) and 10 males (45.46%), with a mean age of 65 ± 10 years. Baseline mean BMI was 26.6 ± 5.0 kg/m^2^, with clinically relevant nutritional risk in 17% of patients (PG‐SGA ≥ 4) and probable sarcopenia in 25% of patients, based on reduced handgrip strength values—no patients presented very high PG‐SGA scores suggestive of severe nutritional risk. A decreased appetite was observed in 8% of patients, and dysphagia in 4%. The anthropometric parameters and blood chemistry tests are shown in Table 1.

**TABLE 1:** Baseline (T0) demographic, nutritional, and hematochemical parameters of the study population.

Parameter	Value
Gender	
Male	10 (45.46%)
Female	12 (54.54%)
BMI (kg/m^2^)	26.6 ± 5.0
PG‐SGA	4.4 ± 4
Malnutrition (%)	17%
Reduced muscle strength—probable sarcopenia (%)	25%
Decreased appetite (%)	8%
Dysphagia (%)	4%
Hematochemical parameters	
Glucose (mg/dL)	107 ± 40
Creatinine (mg/dL)	0.83 ± 0.2
Total cholesterol (mg/dL)	192 ± 46
Calcium (mg/dL)	9.5 ± 0.4
Sodium (mmol/L)	141 ± 3
Potassium (mg/dL)	4.2 ± 0.3
Iron (mcg/L)	84 ± 25
Total protein (g/dL)	6.89 ± 0.4
Albumin (g/dL)	4.5 ± 0.3
Red blood cells (10^3^/μL)	4.8 ± 0.7
White blood cells (10^6^/μL)	6.6 ± 2
Hemoglobin (g/dL)	13.8 ± 2

Abbreviations: BMI, body mass index; PG‐SGA, Patient‐Generated Subjective Global Assessment.

Tumor sites were distributed as follows: tongue (50%), upper alveolar ridge (18.18%), oral floor (13.64%), buccal mucosa (9.09%), and hard palate (9.09%). We selected surgical procedures based on the tumor’s location and extent. Three patients (13.64%) underwent pelvectomies, six (27.27%) hemimaxillectomies, two (9.09%) buccal mucosal removals, and 11 (50%) glossectomies (six Type II glossectomies and five Type IIIa glossectomies according to Ansarin’s classification [26]). In 20 cases, a reconstruction with a pedunculated flap was performed, while in two cases, a direct closure was performed (two glossectomies II). An exhaustive list of reconstruction type specifications is presented in Table 2. Precisely, two cases (9.09%) involved the performance of a nasolabial flap (oral floor tumors), one (4.54%) of which was a Zhao flap (FAMMIF, facial artery musculomucosal island flap) [27] (oral floor tumor), two (9.09%) Bichat fat pad flaps (tumors of the cheek mucosa), six (27.27%) temporalis muscle flaps (hemimaxillectomies), and nine (40.91%) Bozola flaps (buccal artery myomucosal flap) [27, 28] (four Type II glossectomies and five Type IIIa glossectomies). The surgical procedure involved cervical lymph node dissection, which was performed unilaterally (UND, unilateral neck dissection) in 19 patients (86.36%) and bilaterally (BND, bilateral neck dissection) in 3 patients (13.64%). Precisely 19 patients underwent selective neck dissection, as the lymph nodes were clinically and radiologically negative (cN0). In only one case was an UND of I–V levels performed, as the patient had laterocervical lymphadenopathy (cN2).

**TABLE 2:** Clinical‐surgical characteristics of the enrolled patients.

Category	Tumor site	Tumor removal	Neck dissection	Reconstruction
1	Oral floor (n.3, 13.64%)	Pelvectomy with safe margins (n.3)	Yes, UND levels I–III (n.3, 13.64%)	Nasolabial flap (n.2, 9.09%) Zhao flap (n.1, 4.54%)

2	Hard palate (n.2, 9.09%)	Hemi‐maxillectomy (n.2)	Yes, UND levels I–III (n.1, 4.54%)	Temporalis muscle flap (n.2, 9.09%)

3	Buccal mucosa (n.2, 9.09%)	Tumorectomy with safe margins (n.2)	Yes, UND levels I–III (n.2, 9.09%)	Bichat fat pad flap (n.2, 9.09%)

4	Upper alveolar ridge mucosa (n.4, 18.18%)	Hemi‐maxillectomy (n.4)	Yes, UND levels I–III (n.3, 13.64%) Yes, BND levels I–III (n.1, 4.54%)	Temporalis muscle flap (n.4, 18.18%)

5	Tongue (n.11, 50%)	Type II glossectomy (n.6) Type IIIa glossectomy (n.5)	Yes, UND levels I–III (n.9, 40.91%) Yes, lymph node levels I–IV (n.1, 4.54%) Yes, BND levels I–III (n.1, 4.54%)	Direct closure (n.2, 9.09%) Bozola flap (n.9, 40.91%)

Abbreviations: BND, bilateral neck dissection; UND, unilateral neck dissection.

All patients received nasogastric feeding for a mean duration of 7.7 ± 3.1 days and were provided with immunonutrition. On average, patients consumed approximately five bricks during their hospital stay. In the first month after surgery, it was estimated that patients consumed approximately 123 bricks as outpatients. However, adherence was not formally quantified at the individual patient level, and no dose–response analysis between immunonutrition intake and clinical outcomes was performed.

Body weight decreased significantly during the early postoperative period and partially recovered thereafter (Figure 1). Mean weight loss between T1 and T2 was −2.1 kg (SD 3.3; 95% CI −3.4 to −0.8; *p* < 0.001), corresponding to a moderate effect size (Cohen’s *d* = 0.64). This was followed by a partial recovery of +1.2 kg from T2 to T3 (95% CI 0.3 to 2.1; *p* = 0.013).

**FIGURE 1:**
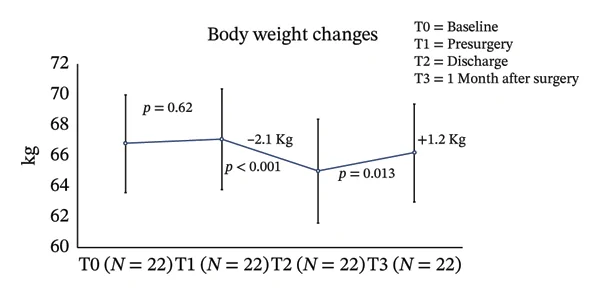
Changes in body weight during the perioperative period in patients with OSCC. Data are presented as mean values; error bars represent the standard error of the mean (SEM).

Total body water decreased significantly between T0 and T1 (−1.8 ± 0.6 L; 95% CI −2.5 to −1.1; *p* = 0.004) and was normalized by T3 (Figure 2).

**FIGURE 2:**
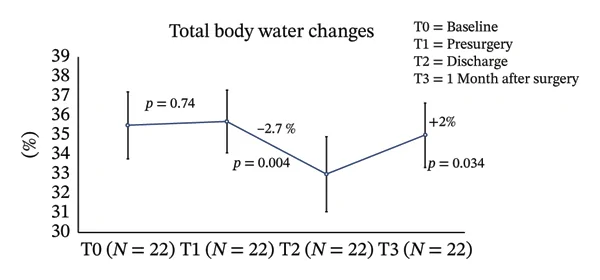
Changes in total body water during the perioperative period in patients with OSCC. Data are presented as mean values; error bars represent the standard error of the mean (SEM).

A significant reduction in muscle mass was observed between T1 and T2 (−2.7 kg; 95% CI −5.3 to −0.1; *p* = 0.045), followed by a significant recovery from T2 to T3 (+1.8 kg; 95% CI 0.5 to 3.1; *p* = 0.010) (Figure 3). The graph shows that muscle mass remained relatively stable before the postoperative decline, followed by a significant loss immediately after surgery, with a significant recovery within 1 month. This pattern is consistent with the expected effects of surgical stress, postoperative catabolism, progressive nutritional recovery, and restoration of oral intake during follow‐up. However, given the observational design and the absence of detailed quantitative dietary intake analysis, these findings cannot be directly attributed to immunonutrition alone.

**FIGURE 3:**
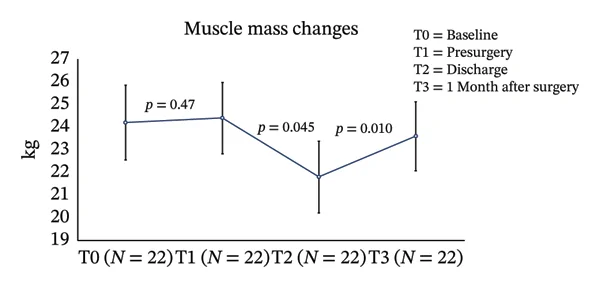
Changes in muscle mass during the perioperative period in patients with OSCC. Data are presented as mean values; error bars represent the standard error of the mean (SEM).

The functional parameters demonstrated stability throughout the follow‐up period. Figure 4 shows a slight but steady decline in handgrip strength over time. From T0 to T1, there is a decrease of about 0.3 kg. A more significant decrease of 0.8 kg is observed from T1 to T2. Instead, from T2 to T3, the force stabilizes. Although a decreasing trend in handgrip strength was noted from baseline until discharge, the *p*‐values (all > 0.05) suggest that these changes were not statistically significant in the analyzed sample.

**FIGURE 4:**
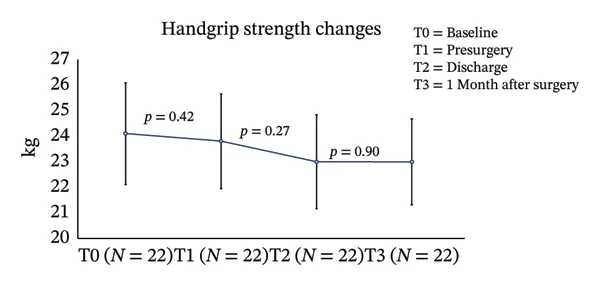
Changes in handgrip strength during the perioperative period in patients with OSCC. Data are presented as mean values; error bars represent the standard error of the mean (SEM).

A similar situation applies to the phase angle: Figure 5 shows a progressive reduction in its average value. From T0 to T1, the value drops from approximately 5.1° to 4.9°. A further slight decrease is then observed from T1 to T2, reaching 4.8° at discharge. This value then remains stable up to T3, indicating a plateau in recovery. These findings suggest that perioperative immunonutrition in patients undergoing surgery for OSCC may be associated with favorable postoperative recovery patterns, despite the expected early postoperative weight loss.

**FIGURE 5:**
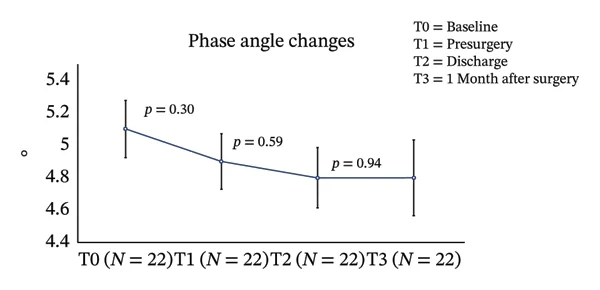
Changes in phase angle during the perioperative period in patients with OSCC. Data are presented as mean values; error bars represent the standard error of the mean (SEM).

The observed wound dehiscence rate (40.91%) appears relatively high at first inspection; however, this finding should be interpreted within the context of major head and neck oncologic surgery requiring extensive intraoral reconstruction. The most common dehiscence events were partial and were clinically manageable. These resolved either with minor resuturing (13.64%) or secondary intention healing (27.27%), without flap loss, fistula formation, or major infectious complications. Research conducted to date about OSCC surgery has reported a range of wound complications, depending on factors such as tumor location, the extent of the surgical procedure, the reconstructive technique used, the patient’s nutritional status, and prior treatment exposure. In this context, the absence of major flap‐related complications in our cohort may suggest a potentially beneficial effect of perioperative nutritional support on tissue healing and postoperative recovery. However, it should be noted that causal inferences cannot be established from the present study design. Transient marginal mandibular nerve palsy occurred in 3 patients (13.64%), and combined facial/accessory nerve dysfunction in 1 patient (4.54%). All patients exhibited recovery in nervous function. The three patients with one‐nerve‐only palsy demonstrated a recovery within 30 days following corticosteroid (betamethasone 4 mg, administered twice daily for an average of 7 days) and neurotrophic (one tablet daily for an average of 20 days) therapy. In the instance of combined paralysis, physiotherapy was also necessary as a therapeutic intervention. No instances of major infections, fistulas, or flap failures were documented. Fifteen patients (68%) received postoperative antibiotics for a duration exceeding 5 days. No deaths occurred within 30 days.

The average LOS was 13.8 ± 6.9 days (95% CI 11.0–16.6). This value appears to be lower than the LOS values previously reported in heterogeneous OSCC surgical cohorts from the literature. However, a direct comparison is not possible due to differences in study populations, perioperative protocols, and institutional management strategies. No correlation was observed between baseline nutritional status and LOS (*r* = 0.21, *p* = 0.34). However, it is important to interpret these findings cautiously due to the limited sample size and observational design.

## 4. Discussion

OSCC is frequently associated with preoperative malnutrition, driven by tumor‐related dysphagia, pain, and catabolic changes, which have been shown to negatively affect surgical outcomes and overall prognosis. Patients suffering from malnutrition and diagnosed with OSCC face an elevated risk of postoperative complications, impaired wound healing, prolonged hospital stays, and diminished quality of life [16, 29, 30]. In this context, perioperative nutritional strategies—particularly immunonutrition—have gained increasing attention as a potential means of mitigating these risks and improving recovery [10, 11, 19]. The present study contributes to the expanding corpus of evidence on this subject by evaluating the effects of perioperative immunonutrition in patients with OSCC undergoing surgery.

The results of this prospective observational cohort study demonstrate that patients undergoing surgery for OSCC who received perioperative immunonutrition experienced limited early postoperative weight loss and favorable clinical trends, including a relatively short hospital stay. Despite an initial decline in total body water and muscle mass during the early postoperative period, functional measures, including handgrip strength and phase angle, demonstrated stability throughout the subsequent follow‐up period. This finding indicates the preservation of functional nutritional reserves, despite transient tissue‐level alterations.

The early postoperative reduction in body weight and muscle‐related parameters observed in our cohort is consistent with the expected catabolic response following major head and neck oncologic surgery. In clinical practice, the recovery of muscle mass and functional nutritional status generally requires prolonged nutritional support and rehabilitation over several weeks or months, particularly in patients with pre‐existing nutritional impairment. Therefore, the modest short‐term recovery observed during follow‐up should not be interpreted as a direct anabolic effect of immunonutrition alone. Instead, perioperative immunonutrition is designed to regulate inflammatory and immune responses, promote wound healing, and enhance tolerance to surgical stress.

Furthermore, achieving postoperative caloric and protein targets is often a gradual process for patients undergoing major oral cavity surgery, particularly during the transition from enteral to oral feeding. Consequently, some degree of early postoperative weight loss is clinically expected despite nutritional support.

Several randomized trials and systematic reviews in the fields of surgical oncology and head and neck cancer have reported results broadly in line with our findings. Meta‐analyses and high‐quality trials consistently demonstrate that perioperative immunonutrition, defined as nutritional formulations enriched with arginine, omega‐3 fatty acids, and nucleotides, exhibits a substantial reduction in the incidence of postoperative infectious complications and surgical‐site infections. In certain clinical series, it has been observed to lead to a decrease in overall postoperative morbidity and anastomotic/suture failures. Furthermore, meta‐analyses indicate that this nutritional intervention results in a mean reduction of approximately 1–3 days in the duration of hospital stay. The protective effects in question have been observed across cohorts of patients undergoing gastrointestinal and head and neck cancer surgery. This observation is consistent with the hypothesis that immune‐modulating nutrients are associated with modified host defenses, wound healing, and recovery pathways after major resection [31].

In the context of head and neck cancer, several randomized and controlled studies (including multicenter and double‐blind trials) have demonstrated a reduction in infectious complications and a tendency toward decreased overall complication rates following perioperative immunonutrition, particularly when the intervention encompassed both preoperative and postoperative phases. The relatively short average LOS observed in our cohort (mean: 13.8 ± 6.9 days) in comparison to the reported values for non‐immunonutrition cohorts is consistent with the findings in the extant literature. However, it is crucial to emphasize that this observation is purely descriptive in nature. The absence of a randomized, concurrent parallel control group renders the claim of a net percentage reduction in hospital admissions mathematically inappropriate. The shorter LOS observed dynamically reflects a combination of early mobilization, structured prehabilitation, and multidisciplinary care, rather than being attributable solely to the immunonutritional protocol. Consequently, any parallels drawn with historical cohorts remain purely hypothesis‐generating and must be interpreted with extreme caution due to critical variations in patient selection, extensive reconstructive surgical techniques, and institutional discharge criteria.

This finding aligns with evidence from systematic reviews and meta‐analyses showing that perioperative immunonutrition is associated with a significant reduction in postoperative complications and hospital LOS across gastrointestinal and OSCC populations [31–33]. In particular, randomized and prospective studies have demonstrated that immunonutrition reduces infectious morbidity and modulates immune and inflammatory responses, thereby facilitating faster postoperative recovery and earlier discharge [34, 35]. These benefits are especially relevant in head and neck cancer patients, who frequently experience nutrition‐impact symptoms related to surgery, chemotherapy, and radiotherapy that can otherwise prolong hospitalization and delay functional recovery [36]. Collectively, the available literature supports the notion that immunonutrition contributes not only to improved clinical outcomes but also to more efficient resource utilization, consistent with the reduced LOS observed in our cohort.

The transient early reductions in total body water and muscle mass that were observed—followed by partial recovery by 1 month—are consistent with findings from other perioperative studies, which highlight the occurrence of immediate postoperative catabolism and fluid shifts [30, 31]. Notably, the maintenance of handgrip strength and stable phase angle in our cohort indicates that the functional consequences of this transient tissue loss were minimal. This suggests that the tissue loss did not have a significant impact on function, potentially due to the preservation of functional status, despite transient postoperative catabolism and multifactorial postoperative recovery mechanisms, including nutritional support.

This functional preservation is consistent with previous reports indicating that maintenance of nutritional status and enhanced tolerance to treatment in head and neck cancer patients receiving enriched formulas [29].

From a mechanistic perspective, immunonutrients such as arginine, omega‐3 fatty acids (EPA/DHA), and nucleotides modulate inflammatory cascades, neutrophil and T‐cell function, and eicosanoid pathways. These mechanisms have the potential to reduce excessive postoperative inflammation and promote wound healing. These mechanisms help explain the consistent signal across the literature for reduced infectious complications and improved wound outcomes [37–41].

A relatively high proportion of patients (68%) received postoperative antibiotic therapy for more than 5 days. This finding is probably indicative of the complexity of major clean‐contaminated head and neck oncologic procedures involving mucosal resection and flap reconstruction, where prolonged antibiotic prophylaxis or empirical treatment is frequently adopted in clinical practice. However, prolonged antibiotic exposure may influence inflammatory and immune responses and could represent a potential confounding factor when interpreting postoperative recovery outcomes. This aspect should be more systematically addressed in future controlled studies.

It should be noted that this study has several limitations. The study’s limitations, including its small sample size, single‐center design, and lack of randomization, restrict its generalizability and the strength of its causal inferences. Furthermore, the presence of heterogeneity in tumor sites and surgical procedures serves to limit the efficacy of direct comparison. These warnings are consistent with the concerns identified in systematic reviews that have detected variability in dosage, timing (preoperative vs. perioperative), baseline nutritional status, and formula composition as sources of heterogeneity across studies. While our findings are encouraging and align with an expanding body of evidence, definitive confirmation necessitates the execution of multicenter randomized clinical trials with standardized immunonutrition protocols and predefined clinical endpoints. A further limitation is the absence of a contemporaneous control group. Therefore, when making comparisons with historical cohorts from the literature, these should be interpreted with caution, as differences in surgical techniques, perioperative care pathways, patient selection, and institutional practice may have influenced postoperative outcomes, including LOS. Therefore, it is not possible to make definitive causal inferences regarding the specific effect of immunonutrition from the present study design. A further key limitation is the exclusion of patients with severe malnutrition at baseline. This criterion was adopted to ensure the feasibility and safety of the perioperative nutritional protocol and to reduce potential confounding related to advanced nutritional deterioration. However, this choice may have introduced selection bias by preferentially including patients with relatively preserved nutritional status and functional reserve. It is important to note that the observed postoperative outcomes and the apparent benefits associated with immunonutrition may not be generalizable to severely malnourished OSCC populations. These populations often represent a clinically vulnerable subgroup with a higher risk of complications. In addition, the limited sample size had a significant impact on the statistical power for subgroup and complication‐based analyses. This limitation restricted the ability to identify associations between immunonutrition exposure and specific postoperative complications.

Future studies should specifically evaluate the role of perioperative immunonutrition in patients with advanced nutritional impairment, who may potentially derive the greatest benefit from targeted nutritional interventions.

### 4.1. Future Challenges and Recommendations

In the future, several challenges must be addressed to translate these preliminary findings into standardized clinical guidelines for oral oncology. A primary challenge is the systematic quantification of patient compliance and adherence to nutritional protocols, particularly during the postdischarge outpatient phase. This necessity requires dedicated multiprofessional surveillance networks. Addressing the confounding impact of prolonged empirical antibiotic use on the gut microbiome and subsequent systemic inflammatory response represents a critical methodological obstacle for future research. In light of the observations made, it is recommended that future research endeavors shift toward large‐scale multicenter randomized controlled trials. These trials should be meticulously designed with sufficient statistical power to assess dose–response relationships and specific clinical endpoints. Examples of such endpoints include flap‐specific complication rates and long‐term disease‐free survival. Finally, the development of targeted protocols is imperative to evaluate the efficacy of intensive immunonutritional prehabilitation in subgroups of severely malnourished and advanced OSCC patients. These patients are currently excluded from standard observational protocols but may derive the most significant clinical benefit from such interventions.

## 5. Conclusions

In conclusion, patients undergoing surgery for OSCC who received perioperative immunonutrition exhibited favorable postoperative recovery patterns and relatively short hospital stays. The present findings offer preliminary observational evidence regarding the integration of structured nutritional protocols into perioperative care pathways for oral cancer. However, further confirmation is required through randomized controlled trials.

## Author Contributions

Conceptualization, T. Montalcini and M. G. Cristofaro; methodology, F. Ferragina and A. R. Sottile; software, R. Pujia and A. Arrotta; validation, T. Montalcini and M. G. Cristofaro; formal analysis, F. Ferragina and Y. Ferro; investigation, F. Ferragina and Y. Ferro; resources, I. Barca; data curation, F. Ferragina and Y. Ferro; writing–original draft preparation, F. Ferragina; writing–review and editing, F. Ferragina and Y. Ferro; visualization, M. G. Cristofaro; supervision, M. G. Cristofaro; project administration, F. Ferragina and I. Barca; funding acquisition, T. Montalcini and M. G. Cristofaro.

## Funding

This research received no external funding.

Open access publishing was facilitated by Universita degli Studi Magna Graecia di Catanzaro, as part of the Wiley–CRUI‐CARE agreement.

## Disclosure

All authors have read and agreed to the published version of the manuscript.

## Ethics Statement

This study was conducted following the ethical standards outlined in the Helsinki Declaration on Medical Protocol and Ethics. The study was approved by the Ethics Committee of Magna Graecia University of Catanzaro (protocol no. 278, dated 2024), and all patients provided informed consent to participate in the study.

## Consent

Informed consent was obtained from all subjects involved in the study.

## Conflicts of Interest

The authors declare no conflicts of interest.

## Supporting Information

Additional supporting information can be found online in the Supporting Information section.

## Supporting information


**Supporting Information** The graphical abstract is intended to provide a concise representation of the study’s design, encompassing the perioperative immunonutrition protocol, the primary clinical outcomes, and the postoperative recovery patterns observed in patients who underwent surgical intervention for oral squamous cell carcinoma.

## Data Availability

Data will be available on request due to privacy/ethical restrictions.
